# Checkpoint inhibitor effectiveness after corticosteroids and second-line immunosuppressants for immune-related adverse events in non-small-cell lung cancer

**DOI:** 10.1016/j.esmoop.2025.106052

**Published:** 2026-01-20

**Authors:** E.J. van Dijk, M.M. Smeenk, F. Bensch, A.M. Sadowksa, M.V. Verschueren, M. Verhaert, A. Llobell, Y. Suzuki, D.F.L. Liew, A. Takeji, A.T.J. Maria, B.J.M. Peters, J.S. Garcia Morillo, K. Chatzidionysiou, M. Lidar, E.C. van der Hout, S. Aspeslagh, G.J.M. Herder, E.M.W. van de Garde, L.E.L. Hendriks, L.B.M. Hijmering-Kappelle, W.S.M.E. Theelen, K.P.M. Suijkerbuijk, R.J. Verheijden

**Affiliations:** 1 Department of Medical Oncology, University Medical Center Utrecht, Utrecht University, Utrecht, The Netherlands; 2 Department of Thoracic Oncology, Netherlands Cancer Institute, Amsterdam, The Netherlands; 3 Department of Pulmonary Diseases, University Medical Center Groningen, University of Groningen, Groningen, The Netherlands; 4 Department of Pulmonary Diseases, GROW—Research Institute for Oncology and Reproduction, Maastricht University Medical Center+, Maastricht University, Maastricht, The Netherlands; 5 Department of Clinical Pharmacy, St. Antonius Hospital, Utrecht/Nieuwegein, The Netherlands; 6 Division of Pharmacoepidemiology and Clinical Pharmacology, Utrecht Institute for Pharmaceutical Sciences, Utrecht University, Utrecht, The Netherlands; 7 Department of Medical Oncology, Vrije Universiteit Brussel (VUB), Universitair Ziekenhuis Brussel (UZ Brussel), Brussels, Belgium; 8 Department of Rheumatology, Institut d’Investigació i Innovació Parc Taulí (I3PT-CERCA), Parc Taulí University Hospital, Universitat Autònoma de Barcelona, Sabadell, Spain; 9 Department of Rheumatology and Nephrology, Japanese Red Cross Fukui Hospital, Fukui, Japan; 10 Department of Rheumatology, Austin Health, Melbourne, Australia; 11 Department of Medicine, University of Melbourne, Melbourne, Australia; 12 Department of Rheumatology, Kanazawa University Hospital, Kanazawa, Japan; 13 Department of Internal Medicine, CHRU de Montpellier, Montpellier, France; 14 Unidad de Enfermedades Autoimmunes Sistemicas y Raras del Adulto, UGC Medicina Interna, Hospital Universitario Virgen del Rocío, Sevilla, Spain; 15 Rheumatology Unit, Department of Medicine Solna, Karolinska Institute, Stockholm, Sweden; 16 Rheumatology Unit, Sheba Medical Center, Tel HaShomer, Israel; 17 Department of Pulmonology, University Medical Center Utrecht, Utrecht, The Netherlands; 18 Department of Pulmonology, Meander Medical Center Amersfoort, Amersfoort, The Netherlands; 19 Department of Epidemiology and Health Economics, Julius Center for Health Sciences and Primary Care, University Medical Center Utrecht, Utrecht University, Utrecht, The Netherlands

**Keywords:** immunosuppression, corticosteroids, checkpoint inhibitor, NSCLC, immune-related adverse events, toxicity

## Abstract

**Background:**

Recent studies have demonstrated that high-dose corticosteroids and second-line immunosuppressants may impair survival of patients with immune-related adverse events (irAEs) upon treatment with immune checkpoint inhibitors (ICIs). In non-small-cell lung cancer (NSCLC), this has not been studied in sufficiently powered studies. This study assessed the association of corticosteroid dose and second-line immunosuppressants for irAEs with survival outcomes in patients with stage IV NSCLC treated with ICIs.

**Patients and methods:**

Patients with stage IV NSCLC treated with ICI-based regimens between 2015 and 2022 in the first or second line who received systemic immunosuppressants for irAEs were retrospectively included from 17 hospitals in eight countries worldwide. Associations of corticosteroid peak and cumulative dose, and use of second-line immunosuppressants with overall survival (OS), cancer-specific survival (CSS), and progression-free survival (PFS) were assessed using Cox proportional hazards regression.

**Results:**

Of 419 included patients, 339 (80.9%) were treated with corticosteroids only and 80 (19.1%) received second-line immunosuppressants. Higher corticosteroid peak dose was associated with impaired OS [adjusted hazard ratio (HR_adj_) 1.63, 95% confidence interval (CI) 1.27-2.08 for an increase by 80 mg prednisolone equivalent] and CSS (HR_adj_ 1.54, 95% CI 1.13-2.08), but not PFS (HR_adj_ 1.05, 95% CI 0.75-1.48). The cumulative corticosteroid dose and use of second-line immunosuppressants were not associated with survival.

**Conclusion:**

Treatment with high corticosteroid peak dose for irAEs is associated with impaired survival in patients with stage IV NSCLC receiving ICI. Treatment of severe irAEs often requires high doses of corticosteroids. However, clinicians should weigh the unfavorable effects of high peak doses against the need for rapid irAE management.

## Highlights

• Previous studies suggest that immunosuppressive treatment of irAEs impairs survival in patients treated with ICI.
• The effect of immunosuppression for irAEs on survival has not been studied in large NSCLC populations.
• In this study, treatment with high steroid peak doses for irAEs was associated with impaired OS and CSS in stage IV NSCLC.
• The cumulative steroid dose and use of second-line immunosuppression were not associated with impaired survival.
• This suggests considerate steroid use when treating irAEs, weighing benefits against potential detrimental survival effects.

## Introduction

Treatment with immune checkpoint inhibitors (ICIs), including antibodies against programmed cell death protein 1 (anti-PD-1) or its ligand (anti-PD-L1), and anti-cytotoxic T lymphocyte-associated protein-4 (anti-CTLA-4), has become the standard of care for advanced non-small-cell lung cancer (NSCLC).^1^ Phase III trials have shown significantly improved survival with ICI monotherapy or combined platinum-based regimens with ICI when compared with chemotherapy alone, especially for patients without actionable genomic alterations.^2^, ^3^, ^4^, ^5^, ^6^ However, the immune activation caused by treatment with ICI can result in immune-related adverse events (irAEs). Low-grade irAEs can often be managed by interruption of ICI and sometimes (topical) corticosteroids. For most grade ≥3 irAEs, permanent discontinuation of ICI and high doses of corticosteroid treatment (1-2 mg/kg prednisolone) followed by steroid tapering are recommended in clinical guidelines.^7^^,^^8^ For steroid-refractory or steroid-dependent irAEs, second-line immunosuppression can be administered.

Several studies have demonstrated that irAE occurrence is associated with improved survival, even when adjusting for immortal time bias.^9^ However, it has been suggested that immunosuppression with highly dosed corticosteroids or second-line immunosuppression may impair ICI effectiveness.^10^, ^11^, ^12^, ^13^, ^14^, ^15^, ^16^ These studies were carried out in cohorts of mainly patients with melanoma, but not in large cohorts of patients with NSCLC. Given the differences in ICI response rate, survival outcomes, and available treatment options (e.g. chemotherapy, targeted therapy) between NSCLC and melanoma, it remains unknown whether the results of these observational studies can be translated to NSCLC patients. This study assessed the effects of steroid peak dose and cumulative dose, as well as the use of second-line immunosuppression, on survival in a worldwide real-world cohort of patients with stage IV NSCLC who received immunosuppression for irAEs upon ICI treatment.

## Patients and methods

### Data collection

This global multicenter cohort study retrospectively included patients with stage IV NSCLC treated with ICI-based regimens in the first or second line, who received systemic immunosuppression for irAEs. Topical corticosteroids and glucocorticoid replacement therapy for hypocortisolism were not considered systemic immunosuppressive treatment. Patients were included from 17 hospitals in eight countries (Supplementary Table S1, Figure S1, available at https://doi.org/10.1016/j.esmoop.2025.106052) between 2015 and 2022. Among the Dutch hospitals and the UZ Brussel, patients were identified via electronic patient files and/or hospital pharmacy registrations. Additionally, patients were included from hospitals participating in the ImmunoCancer International Registry. This study was approved by the medical ethical committee not to be subject to the Medical Research Involving Human Subjects Act, and the requirement for informed consent was waived (MREC NedMec 22/977). Local approval was obtained in each hospital when necessary.

Baseline characteristics at the start of ICI, including age, sex, Eastern Cooperative Oncology Group performance status (ECOG PS), disease stage, brain metastases, and use of corticosteroids for indications other than irAEs, were collected from electronic patient files. Details on the first irAE for which a systemic immunosuppressant was administered, consecutive irAEs, and details on immunosuppression were recorded. IrAEs were graded according to the Common Terminology Criteria for Adverse Events version 5.0.^17^ The type of immunosuppression, together with start and stop dates, peak dose of corticosteroids (maximum dose on 1 day), and cumulative corticosteroid dose (the sum of all daily doses) were registered. Corticosteroid doses were calculated in milligram (mg) prednisolone equivalent.^18^ If immunosuppression was stopped but restarted within 42 days, this period of immunosuppression was also summed, to include treatment of irAE flares.

### Outcomes

To account for immortal time bias between the start of ICI and the onset of an irAE, survival was measured from the initiation of immunosuppression. Overall survival (OS) was calculated from the start date of immunosuppression until the date of death due to any cause. Cancer-specific survival (CSS) was calculated from the start date of immunosuppression until the date of death due to NSCLC. Patients who died of non-NSCLC-related causes (such as infections or cardiovascular events) were censored at their date of death. Progression-free survival (PFS) was defined as the time from the start of immunosuppression until the date of physician-defined disease progression or death, whichever occurred first. Patients who remained event free were censored on the date of last follow-up.

### Statistical analysis

OS, CSS, and PFS were visualized using the Kaplan–Meier method with log-rank test. To assess associations of corticosteroid peak dose, corticosteroid cumulative dose, and second-line immunosuppression with survival, adjusted hazard ratios (HR_adj_) were estimated using multivariable Cox proportional hazards regression with adjustment for sex, age, and ECOG PS at start of ICI, presence of brain and/or liver metastases, line of treatment, type of treatment (ICI and ICI + chemotherapy combination treatment), and type of irAE. Because corticosteroid peak dose and irAE grade were correlated (Spearman’s rho 0.4, Supplementary Figure S2, available at https://doi.org/10.1016/j.esmoop.2025.106052), leading to collinearity, irAE grade was not included in the primary analyses. Secondary analyses, including irAE grade as a covariate and restricted to patients with grade 3 irAEs, were conducted. Associations of peak and cumulative doses of corticosteroids were analyzed in separate models because of the strong correlation between them. Associations between peak dose and survival were primarily assessed with peak dose as a continuous variable. We assumed that high methylprednisolone pulse doses result in violation of the linearity assumption and underestimation of the association between corticosteroid peak dose and survival. Therefore, corticosteroid peak doses of >200 mg prednisone equivalent were winsorized to the highest peak dose ≤200 mg for the Cox proportional hazards regression analyses. A sensitivity analysis was carried out including true corticosteroid peak dose while allowing for non-linearity by using restricted cubic splines with three prespecified knots at 40, 80, and 160 mg with visualization of the predicted HR_adj_ for each possible peak dose relative to 40 mg. Because body weight could not be collected for all patients, survival was also compared for corticosteroid peak dose when categorized into <60 mg, 60-100 mg, and >100 mg, as this roughly corresponds to 0.5 mg/kg, 1.0 mg/kg, and 2.0 mg/kg prednisone, respectively (Supplementary Figure S3, available at https://doi.org/10.1016/j.esmoop.2025.106052). To account for immortal time between the start of corticosteroids and second-line immunosuppression, we conducted a time-varying survival analysis, in which the start of second-line immunosuppression was included as a time-varying covariate. Because cumulative corticosteroid dose is likely correlated with the duration of corticosteroid use, and thus survival time, we planned a landmark analysis to minimize immortal time bias. In this analysis, only the subset of patients who were still alive 6 months after the start of ICI and whose corticosteroid treatment did not exceed 6 months were included. Statistical significance was set at *P* < 0.05 (two-sided test).

All analyses were carried out using R version 4.4.1.

## Results

In total, 419 patients were included. The mean age of patients was 65 years, 90.7% of patients had an ECOG PS of 0 or 1, and 62.5% were treated with ICI in the first line (Table 1). Most patients received anti-PD-1-based treatment (*n* = 393, 93.8%), 9 patients (2.1%) received anti-PD-L1, and 17 patients (4.0%) received one of these together with anti-CTLA-4. The majority received ICI only (65.6%), and 144 patients (34.4%) received ICI in combination with chemotherapy. Only a small number of patients (*n* = 27, 6.4%) received corticosteroids at baseline.

**Table 1:** Baseline characteristics at the time of start of immune checkpoint inhibition

	Corticosteroids only (*n* = 339)	Second-line immunosuppression (*n* = 80)	Overall (*N* = 419)
Sex, *n* (%)			
Female	153 (45.1)	45 (56.3)	198 (47.3)
Male	186 (54.9)	35 (43.8)	221 (52.7)
Age, years			
Mean (SD)	65.3 (9.8)	65.0 (9.2)	65.2 (9.6)
ECOG PS, *n* (%)			
0-1	313 (92.3)	67 (83.8)	380 (90.7)
2-4	21 (6.2)	9 (11.3)	30 (7.2)
Missing	5 (1.5)	4 (5.0)	9 (2.1)
Known brain metastases, *n* (%)			
Yes	61 (18.0)	16 (20.0)	77 (18.4)
No	277 (81.7)	64 (80.0)	341 (81.4)
Missing	1 (0.3)	0 (0)	1 (0.2)
Known liver metastases, *n* (%)			
Yes	34 (10.0)	9 (11.3)	43 (10.3)
No	304 (89.7)	71 (88.8)	375 (89.5)
Missing	1 (0.3)	0 (0)	1 (0.2)
Type of ICI, *n* (%)			
Anti-PD-1	319 (94.1)	74 (92.5)	393 (93.8)
Anti-PD-1 and anti-CTLA-4	12 (3.5)	4 (5.0)	16 (3.8)
Anti-PD-L1	7 (2.1)	2 (2.5)	9 (2.1)
Anti-PD-L1 and anti-CTLA-4	1 (0.3)	0 (0)	1 (0.2)
Concurrent therapy, *n* (%)			
None	226 (66.7)	49 (61.3)	275 (65.6)
Chemotherapy	113 (33.3)	31 (38.8)	144 (34.4)
Line of treatment, *n* (%)			
1	203 (59.9)	59 (73.8)	262 (62.5)
2	136 (40.1)	21 (26.3)	157 (37.5)
Previous systemic treatment,^a^*n* (%)			
Platinum-containing chemotherapy	125 (91.9)	19 (90.5)	144 (91.7)
BRAF/MEK inhibition	3 (2.2)	0 (0.0)	3 (1.9)
Different checkpoint inhibitor	2 (1.5)	0 (0.0)	2 (1.3)
Other	1 (0.7)	1 (4.8)	2 (1.3)
Missing	5 (1.2)	1 (4.8)	6 (3.8)
PD-L1 expression,^b^*n* (%)			
<1%	91 (26.8)	21 (26.3)	112 (26.7)
1%-49%	63 (18.6)	17 (21.3)	80 (19.1)
≥50%	124 (36.6)	29 (36.3)	153 (36.5)
Missing	61 (18.0)	13 (16.3)	74 (17.7)
Baseline corticosteroid use, *n* (%)			
Yes	21 (6.2)	6 (7.5)	27 (6.4)
No	317 (93.5)	73 (91.3)	390 (93.1)
Missing	1 (0.3)	1 (1.3)	2 (0.5)
Grade of irAE, *n* (%)			
1	6 (1.8)	1 (1.3)	7 (1.7)
2	113 (33.3)	23 (28.8)	136 (32.5)
3	187 (55.2)	44 (55.0)	231 (55.1)
4	24 (7.1)	11 (13.8)	35 (8.4)
5	6 (1.8)	0 (0)	6 (1.4)
Missing	3 (0.9)	1 (1.3)	4 (1.0)
Type of irAE, *n* (%)			
Cardiac	4 (1.2)	1 (1.3)	5 (1.2)
Cutaneous	23 (6.8)	3 (3.8)	26 (6.2)
Endocrine	3 (0.9)	0 (0)	3 (0.7)
Gastrointestinal	73 (21.5)	29 (36.3)	102 (24.3)
Hepatobiliary	56 (16.5)	14 (17.5)	70 (16.7)
Neuromuscular	16 (4.7)	6 (7.5)	22 (5.3)
Rheumatic	20 (5.9)	11 (13.8)	31 (7.4)
Pulmonary	107 (31.6)	7 (8.8)	114 (27.2)
Renal	19 (5.6)	2 (2.5)	21 (5.0)
Other	18 (5.3)	7 (8.8)	25 (6.0)

CTLA-4, cytotoxic T lymphocyte-associated protein-4; ECOG PS, Eastern Cooperative Oncology Group performance status; ICI, immune checkpoint inhibitor; irAE, immune-related adverse event; PD-1, programmed cell death protein 1; PD-L1, programmed death-ligand 1; SD, standard deviation; TGF, transforming growth factor.

a Percentage calculated only among patients treated with ICI in the second line. First-line checkpoint inhibitors in these patients included ipilimumab + nivolumab, and anti-PD-L1 therapy combined with an anti-TGF-β agent in clinical trials. Other first-line treatments included capecitabine/oxaliplatin/bevacizumab in a patient with a concurrent cecal carcinoma, and tegafur/gimeracil/oteracil in one patient.

b Per hospital protocol.

### Immune-related adverse events and immunosuppressants

Immunosuppression was most often started for pulmonary (27.2%), gastrointestinal (24.3%), or hepatobiliary (16.7%) irAEs. The majority of patients (54.4%) were treated for grade 3 irAEs. Most patients (*n* = 339, 80.1%) received only corticosteroids, and 80 patients (19.1%) required second-line immunosuppression such as tumor necrosis factor inhibition (*n* = 32, 7.6%), mycophenolate mofetil (*n* = 28, 6.7%), or calcineurin inhibitors (*n* = 9, 2.1%, mainly tacrolimus). Sixteen patients received more than one type of second-line immunosuppression (Supplementary Figure S4, available at https://doi.org/10.1016/j.esmoop.2025.106052). Compared with patients receiving second-line immunosuppression, corticosteroid-only patients more often received ICI in the second line (40.1% versus 26.3%), more often had pulmonary irAEs (31.6% versus 8.8%), and less frequently had gastrointestinal (21.5% versus 36.3%) and rheumatic (5.9% versus 13.8%) irAEs (Table 1). Eleven patients (2.6%) died due to irAEs, including seven due to pulmonary irAEs.

Median time between start of ICI and onset of the irAE for which immunosuppression was started was 109 days (Q1-Q3 42-256 days), and median time between onset of toxicity and the start of immunosuppression was 2 days (Q1-Q3 1-14 days). Median duration of immunosuppression was 93 days (Q1-Q3 43-202 days). The median peak dose of corticosteroids was 60 mg (Q1-Q3 50-80 mg), and the median cumulative dose of corticosteroids was 2480 mg (Q1-Q3 1418-4668 mg). Only 14 patients (3.3%) received a corticosteroid peak dose >200 mg; these patients received (methyl)prednisolone pulse dosing followed by tapering. For patients who received only corticosteroids, the median peak dose was 60 mg (Q1-Q3 50-80 mg). Patients who received second-line immunosuppression also received a median steroid peak dose of 60 mg (Q1-Q3 60-100 mg). Patients who received second-line immunosuppression were treated for a median of 45 days (Q1-Q3 2-134 days). The median duration of treatment with ICI was 162 days (Q1-Q3 63-378 days).

### Peak dose corticosteroids and survival

Median OS after starting immunosuppression was 14.4 months [95% confidence interval (CI) 12.5-19.3 months]. Median CSS was 25.4 months (95% CI 19.0-36.4 months), and median PFS was 13.3 months (95% CI 11.3-19.8 months). Causes of death mainly included progression of NSCLC (44.6% of total study population), irAEs (2.6%), infections (2.6%), cardiovascular events (2.4%), respiratory failure (1.0%), and other malignancies (1.0%) (Supplementary Table S2, available at https://doi.org/10.1016/j.esmoop.2025.106052).

When comparing patients receiving corticosteroid peak doses of <60 mg, 60-100 mg, and >100 mg (Figure), the median OS was 20.7 months (95% CI 13.4-35.0 months) versus 15.4 months (95% CI 12.1-25.4 months) versus 7.3 months (95% CI 3.7-12.4 months, *P* < 0.001). Median CSS was 29.4 months (95% CI 19.0-62.9 months) versus 30.8 months (95% CI 19.3-46.7 months) versus 11.4 months (95% CI 7.3-35.2 months, *P* = 0.005), and the median PFS was 20.2 months (95% CI 12.6-30.8 months) versus 12.6 months (95% CI 7.0-19.8 months) versus 7.1 months [95% CI 5.9 months-not reached (NR), *P* = 0.40].

**Figure 1:**
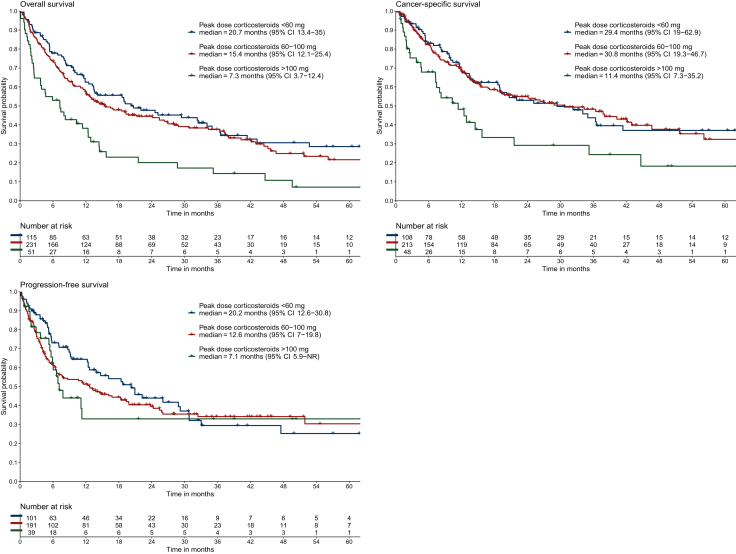
**Overall survival, cancer-specific survival, and progression-free survival as assessed by Kaplan–Meier analyses, measured from start of immunosuppression, stratified for peak dose of corticosteroids.** CI, confidence interval; NR, not reached.

In multivariable analyses, higher peak dose was significantly associated with worse OS (HR_adj_ 1.63, 95% CI 1.27-2.08, for each dose increase by 80 mg prednisolone equivalent) and CSS (HR_adj_ 1.54, 95% CI 1.13-2.08), but not with PFS (HR_adj_ 1.05, 95% CI 0.75-1.48) (Table 2). Sensitivity analyses confirmed these associations or demonstrated similar HR point estimates, including analyses with true corticosteroid peak dose (no winsorization) with allowance of non-linearity (Supplementary Table S3 and Figure S5), excluding patients with progressive disease before the start of immunosuppression (Supplementary Table S4), measuring survival from the start of ICI instead of from the start of immunosuppression (Supplementary Table S5), including irAE grade in the multivariable models (Supplementary Table S6), including only patients with grade 3 irAEs (Supplementary Table S7), and including only patients who did not receive concurrent chemotherapy (Supplementary Table S8, available at https://doi.org/10.1016/j.esmoop.2025.106052).

**Table 2:** Multivariable Cox proportional hazards regression analysis with peak dose corticosteroids and use of second-line immunosuppressants

Variable	HR (95% CI) OS	HR (95% CI) CSS	HR (95% CI) PFS
Peak dose corticosteroids (per 80 mg)	1.63 (1.27-2.08)	1.54 (1.13-2.08)	1.05 (0.75-1.48)
Lines of immunosuppression			
Corticosteroids only	—	—	—
Second-line immunosuppression	0.86 (0.60-1.23)	0.79 (0.50-1.24)	0.73 (0.47-1.12)
Sex			
Female	—	—	—
Male	1.14 (0.88-1.47)	1.25 (0.91-1.72)	0.77 (0.57-1.03)
Age, years	1.03 (1.01-1.04)	1.03 (1.01-1.04)	1.01 (1.00-1.03)
ECOG PS			
0-1	—	—	—
2-4	1.77 (1.12-2.81)	1.84 (0.99-3.41)	1.53 (0.88-2.67)
Brain metastases			
No	—	—	—
Yes	1.04 (0.76-1.44)	0.99 (0.67-1.48)	1.18 (0.80-1.73)
Liver metastases			
No	—	—	—
Yes	1.75 (1.20-2.55)	2.14 (1.38-3.33)	1.21 (0.74-1.99)
Line of treatment			
1	—	—	—
2	1.76 (1.31-2.36)	1.87 (1.29-2.73)	1.41 (0.97-2.05)
Type of treatment			
Anti-PD-(L)1 + chemotherapy	—	—	—
Anti-PD-(L)1 + ipilimumab with or without chemotherapy	0.78 (0.38-1.61)	0.62 (0.24-1.61)	0.90 (0.42-1.92)
Anti-PD-(L)1 monotherapy	0.67 (0.48-0.92)	0.56 (0.38-0.82)	0.64 (0.44-0.92)
Type of irAE			
Gastrointestinal	—	—	—
Hepatobiliary	1.16 (0.77-1.76)	1.02 (0.60-1.72)	1.28 (0.79-2.08)
Other	0.96 (0.68-1.37)	0.92 (0.60-1.41)	1.33 (0.89-1.98)
Pulmonary	1.38 (0.97-1.96)	1.45 (0.95-2.20)	1.31 (0.85-2.00)

CI, confidence interval; CSS, cancer-specific survival; ECOG PS, Eastern Cooperative Oncology Group performance status; HR, hazard ratio; irAE, immune-related adverse event; OS, overall survival; PD-(L)1, programmed death-(ligand) 1; PFS, progression-free survival.

### Cumulative dose corticosteroids and survival

No associations between cumulative corticosteroid dose and survival were found in multivariable analyses for either OS (HR_adj_ 0.99, 95% CI 0.97-1.02, per 1000 mg prednisolone equivalent), CSS (HR_adj_ 0.99, 95% CI 0.97-1.02), or PFS (HR_adj_ 1.00, 95% CI 0.99-1.02) (Supplementary Table S9, available at https://doi.org/10.1016/j.esmoop.2025.106052). In the landmark analysis in which patients were only included if they were alive 6 months after the initiation of immunosuppression and received corticosteroids for <6 months, no association of cumulative corticosteroid dose and OS (*n* = 171; HR_adj_ 1.04, 95% CI 0.93-1.16), CSS (*n* = 159; HR_adj_ 1.02, 95% CI 0.90-1.16), or PFS (*n* = 151; HR_adj_ 0.98, 95% CI 0.88-1.11) was found (Supplementary Table S10, available at https://doi.org/10.1016/j.esmoop.2025.106052).

### Second-line immunosuppression and survival

When comparing patients receiving only corticosteroids to patients receiving second-line immunosuppressants, median OS was 13.4 months (95% CI 11.9-19.0 months) versus 21.3 months (95% CI 14.4-30.9 months, *P* = 0.30), median CSS was 21.6 months (95% CI 15.5-36.4 months) versus 26.9 months (95% CI 20.7 months-NR, *P* = 0.11), and median PFS was 12.6 months (95% CI 9.1-18.4 months) versus 26.0 months (95% CI 7.6 months-NR, *P* = 0.13) (Supplementary Figure S6, available at https://doi.org/10.1016/j.esmoop.2025.106052).

In multivariable analyses, use of second-line immunosuppressants was not significantly associated with OS (HR_adj_ 0.86, 95% CI 0.60-1.23), CSS (HR_adj_ 0.79, 95% CI 0.50-1.24), or PFS (HR_adj_ 0.73, 95% CI 0.47-1.12) when adjusting for peak dose steroids (Table 2). Because patients receiving second-line immunosuppression have at least lived long enough to receive this second-line immunosuppression [median 44 days (interquartile range 12-93 days)], immortal time bias could (partly) explain this trend toward favorable survival in this group. Therefore, a time-varying covariate analysis was carried out. Survival was measured for all patients from start of corticosteroids until progression, death, last follow-up, or start of second-line immunosuppression. For patients with second-line immunosuppression, survival was measured from start of second-line immunosuppression until progression, death, or last follow-up. In these analyses, differences were no longer observed in OS (HR_adj_ 1.15, 95% CI 0.79-1.66), CSS (HR_adj_ 1.06, 95% CI 0.66-1.70), and PFS (HR_adj_ 0.99, 95% CI 0.63-1.54) (Supplementary Figure S7 and Table S11, available at https://doi.org/10.1016/j.esmoop.2025.106052).

### Resumption of ICI

ICI was resumed after toxicity and before the occurrence of progressive disease in only 17 patients (4.1%). Additionally, 112 patients (26.7%) were rechallenged with ICI after progressive disease. ICI was resumed/rechallenged in 37.1% of patients after corticosteroid peak doses <60 mg, 28.7% of patients after peak doses between 60 and 100 mg, and 15.7% of patients after peak doses >100 mg. When adjusting for either resumption or resumption/rechallenge of ICI, results were consistent with the primary analyses (Supplementary Tables S12 and S13, available at https://doi.org/10.1016/j.esmoop.2025.106052).

## Discussion

In this multicenter global cohort study, the largest to date in stage IV NSCLC patients treated with immunosuppression for irAEs during ICI treatment, we observed that high peak corticosteroid dose for irAEs was associated with impaired OS and CSS but not PFS. Cumulative dose of corticosteroids and second-line immunosuppression were not significantly associated with survival. These results are largely in line with previous studies assessing the impact of corticosteroid use for irAEs on survival. In two observational melanoma studies, significantly impaired OS was described for patients who were treated for irAEs with high corticosteroid peak doses when compared with lower peak doses (HR_adj_ 1.29 for 80 mg versus 40 mg; HR_adj_ 1.002 for each mg dose increase; both in prednisolone equivalent).^14^^,^^19^ In another melanoma cohort, a steroid peak dose ≥60 mg for early-onset irAEs (<8 weeks after start ICI) was associated with impaired OS (HR_adj_ 1.97) when compared with lower doses.^15^ In trial data of patients with miscellaneous tumor types treated with ipilimumab combined with nivolumab, impaired OS after high steroid peak dose (HR_adj_ 1.21 and HR_adj_ 1.66 for 1 and 2 versus 0.5 mg/kg, respectively) was seen.^13^ In a cohort of patients with severe ICI-induced enterocolitis who also received infliximab, unfavorable survival was reported after high prednisolone doses (≥75 mg daily) when compared with patients with lower prednisolone doses combined with infliximab.^20^

In our analysis, second-line immunosuppression was not associated with impaired survival, in contrast to previous studies. In advanced melanoma patients who received second-line immunosuppression for irAEs, impaired PFS, OS, and CSS were described when compared with patients receiving steroids alone (HR_adj_ for death 1.54, 95% CI 1.03-2.30).^12^ However, this analysis was not adjusted for steroid peak dose. Verheijden et al. also found an association between second-line immunosuppression and impaired survival in advanced melanoma while adjusting for steroid use (HR for death 1.34, 95% CI 0.99-1.82),^14^ and a similar but non-significant association using pooled trial data from patients with various tumor types (HR_adj_ 1.23, 95% CI 0.90-1.68).^13^ In the melanoma study, patients receiving second-line immunosuppression had higher peak prednisone doses (median 110 versus 80 mg).^14^ In our study, the median peak dose was lower (60 mg) and did not differ between patients with and without second-line immunosuppression. Additionally, the time between corticosteroid start and second-line immunosuppression was longer in our cohort (median 44 days) compared with the abovementioned studies (median 11-24 days). This may introduce immortal time bias, as patients must survive long enough to receive second-line immunosuppression. The time-varying covariate analysis we conducted can only partially adjust for this: patients with response to treatment or more indolent disease courses might have been more likely to survive to the point of second-line immunosuppression, which may contribute to the observed favorable outcomes after its initiation.

The observed association between steroid peak dose and survival could be biased by indication, and more severe irAEs likely require higher steroid peak doses. However, when restricting to patients with grade 3 irAEs, results remained consistent. When adjusting for irAE grade, the association between steroid peak dose and survival persisted, albeit less strong, which is a result of collinearity. Furthermore, given the low number of grade 5 irAEs in this cohort (11 patients, 2.6%), it is unlikely that the association between high peak dose corticosteroids and impaired survival reflects irAE-related mortality. However, residual bias by indication cannot entirely be excluded.

Unlike the association with high steroid peak doses, the cumulative dose was not associated with survival. This raises questions about the pharmacological relations between corticosteroid dosing and its immunosuppressive effects. It has been suggested that suppression of lymphocyte proliferation may depend on the maximum glucocorticoid concentration,^21^^,^^22^ but evidence is limited. Clarifying how peak versus cumulative dosing impacts the immune system could help to optimize therapeutic strategies.

Interestingly, OS and CSS were significantly impaired in patients receiving high steroid peak doses, while PFS was not. In our previously published pooled trial data from mixed tumor types, including NSCLC, high peak dose was associated with both worse OS and PFS, though more strongly with OS.^13^ It remains unclear why we did not find an association with PFS in this study. We hypothesized that patients with high peak doses were less likely to be rechallenged with ICI after progression, thereby affecting OS and CSS but not PFS. Yet, adjusting for ICI rechallenge did not alter the results. Nevertheless, patients exposed to high steroid peak doses might have compromised suitability for, or diminished efficacy of, subsequent treatment options after progression on ICI, for instance, through worsened condition. Another explanation for the lack of PFS association in our study could be that disease progression was physician defined rather than centrally assessed. In this real-world, multicenter setting, response assessment may have been less reliable. It is conceivable, for instance, that treating physicians continued treatment for clinical benefit to patients with marginal disease progression, while objective response measurement would have concluded progressive disease, particularly when subsequent treatment options were limited. Lastly, selection bias could also contribute to the discrepancy between OS/CSS and PFS associations, as patients with progression before immunosuppression were excluded from PFS analyses. However, peak dose remained associated with worse OS and CSS when excluding these same patients, or when measuring survival from ICI start.

Our study is the first to report on survival associations with immunosuppressive treatment for irAEs during ICI treatment in a large NSCLC-only cohort. Compared with melanoma patients, those with NSCLC are more likely to have received previous radiotherapy and previous/concurrent chemotherapy, and more often have a history of smoking and smoking-related comorbidities. These factors may influence both the presentation and the clinical course of certain irAEs, including pneumonitis. The pattern of irAEs has indeed been shown to differ between tumor types, with relatively more gastrointestinal and cutaneous irAEs in melanoma, and more pneumonitis in NSCLC populations.^23^ Respiratory irAEs have been described to have high fatality rates.^24^ Also in our study, a high prevalence of pneumonitis was seen (27.2% of the total population), and pulmonary irAEs were non-significantly associated with worse OS (HR_adj_ 1.38, 95% CI 0.97-1.96) and CSS (HR_adj_ 1.45, 95% CI 0.95-2.20) when compared with gastrointestinal irAEs. However, only seven patients (1.7%) died as a direct consequence of pulmonary irAEs in our cohort. The incidence of pneumonitis in our cohort is somewhat higher than that reported in clinical trials assessing anti-PD-1 therapy in stage IV NSCLC (3%-8% of all patients treated with anti-PD-1,^2^, ^3^, ^4^, ^5^^,^^25^ and 19.4%-28.6% of patients with irAEs^3^^,^^5^^,^^25^). However, in the trials, patients were often excluded if they had recent exposure to radiotherapy, a history of interstitial lung disease, or a poor ECOG PS, which may underlie, at least in part, the higher incidence observed in our real-world population.

This multicenter, global study describes the largest cohort of stage IV NSCLC patients with immunosuppression for irAEs to date. However, this research needs to be interpreted in the light of its limitations. Given the observational, retrospective design, residual confounding could have influenced results. Body weight was missing in almost half (44.6%) of the patients, limiting the ability to analyze steroid doses on a per-kilogram basis, and PD-L1 expression was missing in 17.7% of cases. Information on comorbidities and previous radiotherapy was unknown. These variables may have influenced the type and grade of irAEs (for which we have adjusted in the multivariable analyses). Moreover, information on response to prior systemic treatments and tumor burden was missing. However, we consider it unlikely that these factors confound the association between corticosteroid dose and survival directly. The study population was geographically diverse, however, predominantly European-based, potentially reducing generalizability. Lastly, this study included patients who received systemic immunosuppression for irAEs between 2015 and 2022 according to the guidelines at that time, which may have been higher peak doses than currently advised. Based on this study, it remains difficult to conclude on the association of lower corticosteroid doses with survival.

The need for prospective trials remains to determine which immunosuppressive strategies for irAE treatment would least harm ICI efficacy.

### Conclusion

In conclusion, we observed that treatment with high corticosteroid peak doses for irAEs was associated with impaired OS and CSS in patients with stage IV NSCLC, while corticosteroid cumulative dose and second-line immunosuppressants were not. These findings suggest the importance of a considerate approach to corticosteroid use when treating irAEs, weighing their benefits against the possible detrimental effects on survival. Adding second-line immunosuppressive medication early to avoid high corticosteroid peak doses, although not assessed in this study, could be considered as a strategy to investigate in future studies.

## Disclosure

**LELH** received research funding from Roche Genentech, AstraZeneca, Boehringer Ingelheim, Takeda, Merck, Pfizer, Novartis, and Gilead (all to institution); speaker educationals/webinars: AstraZeneca, Bayer, Lilly, MSD, high5oncology, Takeda, Janssen, GSK, Sanofi, Pfizer (all to institution), Medtalks (self), Benecke (self), VJOncology (self), and Medimix (self); advisory boards: AbbVie, Amgen, Anhearth, AstraZeneca, Bayer, BMS, Boehringer Ingelheim, Daiichi, GSK, Janssen, Lilly, Merck, MSD, Novartis, Pfizer, Pierre Fabre, Roche, Sanofi, Summit Therapeutics, and Takeda (all to institution); member guideline committees: Dutch guidelines on NSCLC, brain metastases and leptomeningeal metastases (payment to self), ESMO guidelines on metastatic NSCLC, non-metastatic NSCLC and SCLC (non-financial); other (non-financial): secretary and as of October 2024 chair NVALT studies foundation, subchair EORTC metastatic NSCLC systemic therapy, vice-chair scientific committee Dutch Thoracic Group. local PI of clinical trials: AstraZeneca, GSK, Novartis, Merck, Roche, Takeda, Blueprint, Mirati, AbbVie, Gilead, MSD, Merck, Amgen, Boehringer Ingelheim, and Pfizer (all to institution); all not related to the current manuscript. **WSMET** received research grants to the NKI from MSD, AZ, and Sanofi/Regeneron. **KPMS** has advisory/consultancy relations with AbbVie, Bristol Myers Squibb, and Pierre Fabre Pharmaceuticals; she received research grants from Bristol Myers Squibb, Genmab, Philips, and Tigatx, all paid to institution; she does data and safety monitoring for Sairopa. All other authors have declared no conflicts of interest.
