# Supplementary material for: Checkpoint inhibitor effectiveness after corticosteroids and second-line immunosuppressants for immune-related adverse events in non-small-cell lung cancer
Source: ESMO Open. 2026 Jan 20;11(2):106052. doi: 10.1016/j.esmoop.2025.106052 (PMC12857322; doi:10.1016/j.esmoop.2025.106052)
Supplement: Supplementary Data [file mmc1.pdf]

Supplementary tables and figures to:

Checkpoint inhibitor effectiveness after corticosteroids and second-line immunosuppressants for immune-related adverse events in non-small cell lung cancer

- Supplementary Table S1: Number of included patients per center
- Supplementary Figure S1: Flow diagram of patient inclusions
- Supplementary Figure S2: Peak corticosteroid dose plotted against irAE grade and type
- Supplementary Figure S3: Corticosteroid dose category plotted against body weight
- Supplementary Figure S4: Type and order of second-line immunosuppressants
- Supplementary Table S2: Overview of causes of death of the study population
- Supplementary Table S3: Analysis with true corticosteroid peak dose (non-winsorized data)
- Supplementary Figure S5: Restricted cubic splines model
- Supplementary Table S4: Analysis in subset of patients that was suitable for PFS analyses
- Supplementary Table S5: Survival analyses when measured from the start of ICI therapy
- Supplementary Table S6: Multivariable analyses including irAE grade
- Supplementary Table S7: Analyses in patients with grade 3 irAEs only
- Supplementary Table S8: Analysis in patients with ICI monotherapy
- Supplementary Table S9: Cumulative dose and second-line immunosuppressants
- Supplementary Table S10: Landmark analysis to assess cumulative steroid dose associations
- Supplementary Figure S6: Survival plots stratified for type of immunosuppression
- Supplementary Figure S7: Survival plots including second-line IS as time-varying covariate
- Supplementary Table S11: Time-varying survival analysis
- Supplementary Table S12: Sensitivity analysis adjusting for ICI resumption
- Supplementary Table S13: Sensitivity analysis adjusting for ICI resumption or rechallenge

Supplementary Table S1: Number of included patients per center

Country	Center	Number of included patients
**The Netherlands**	Netherlands Cancer Institute	161
	University Medical Center Groningen	54
	Maastricht University Medical Center+	52
	University Medical Center Utrecht	26
	Catharina Ziekenhuis Eindhoven	22
	Meander Medical Center Amersfoort	20
	St. Antonius Hospital Utrecht/Nieuwegein	17
	Onze Lieve Vrouwe Gasthuis Amsterdam	5
**Belgium**	Universitair Ziekenhuis Brussel	19
**Japan**	Japanese Red Cross Fukui Hospital	14
	Kanazawa University Hospital	3
**Spain**	Hospital Universitari Parc Tauli	14
	Hospital Universitario Virgen del Rocío	2
**Australia**	Austin Hospital	4
**France**	University Hospital of Montpellier	3
**Israel**	Sheba Medical Center	2
**Sweden**	Karolinska Institutet	1

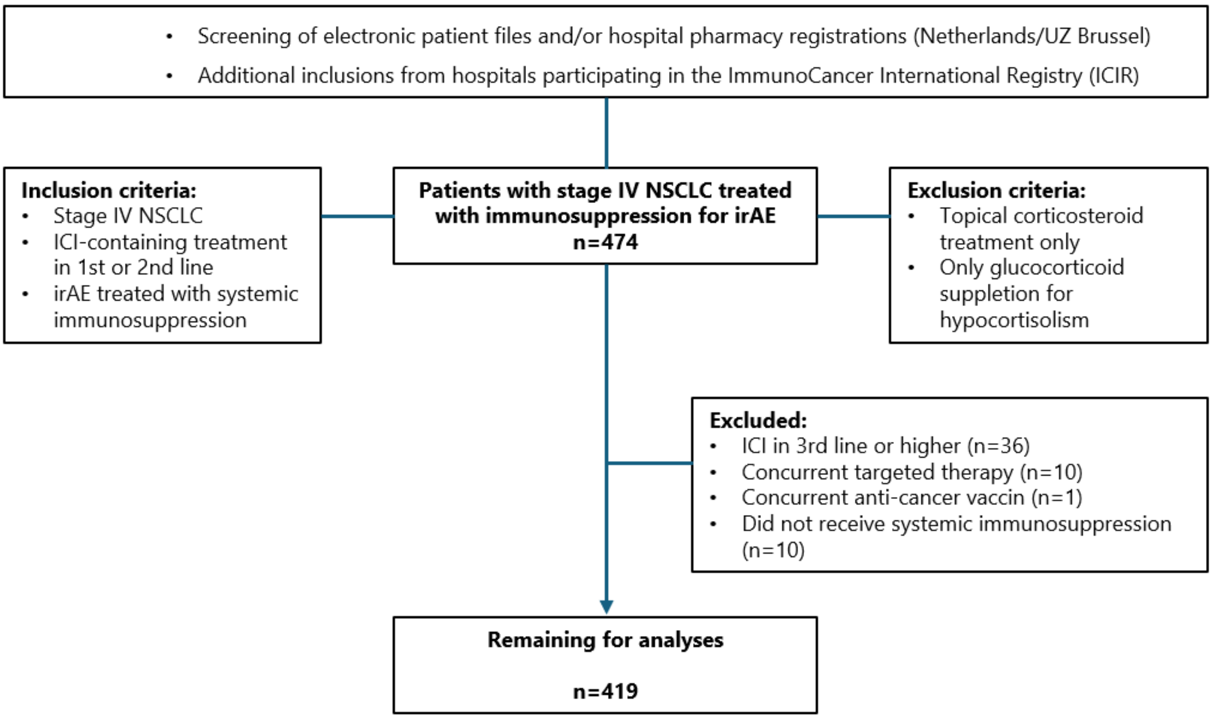

Supplementary Figure S1: Flow diagram of patient inclusions

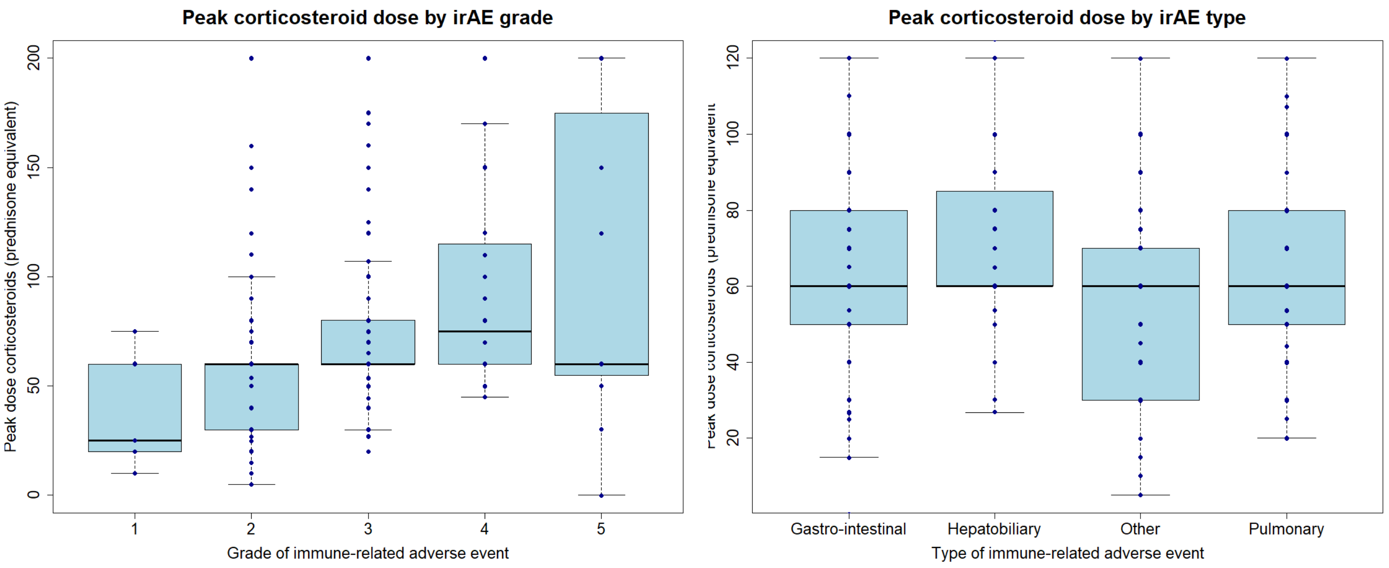

<u>Supplementary Figure S2:</u> <u>Boxplots of peak corticosteroid dose against immune-related adverse event grade and type.</u> Spearman's rank correlation showed a moderate positive association between peak corticosteroid dose and irAE grade (ρ = 0.4, p <0.001).

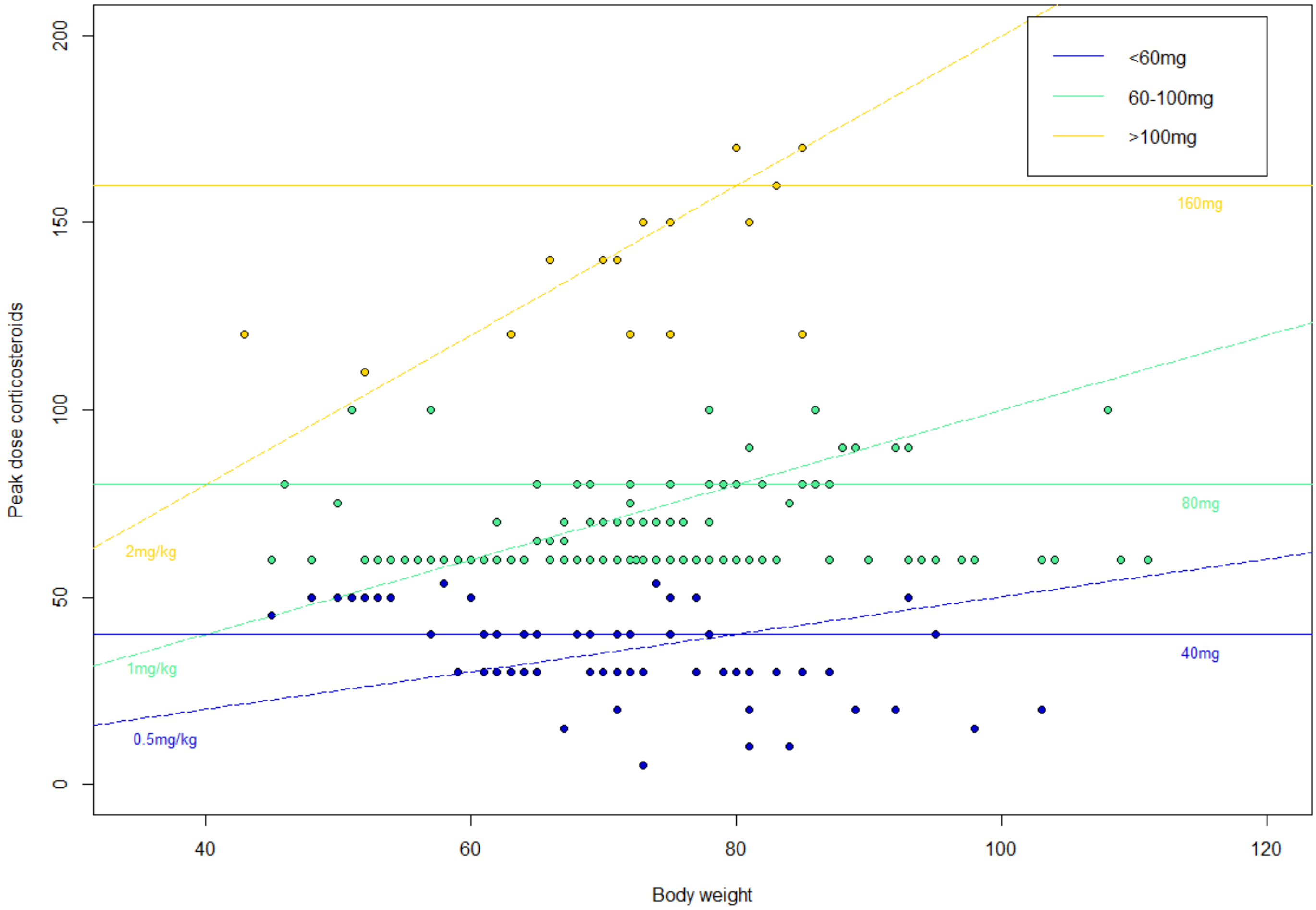

<u>Supplementary Figure S3:</u> <u>Correlation between corticosteroid dose category and body weight.</u> Administered peak dose corticosteroids plotted against body weight, in subgroup of n=232 (55.4%) patients of whom body weight was known. A peak dose of <60 mg roughly corresponds to 0.5mg/kg, 60-100mg to 1mg/kg and >100mg to 2mg/kg.

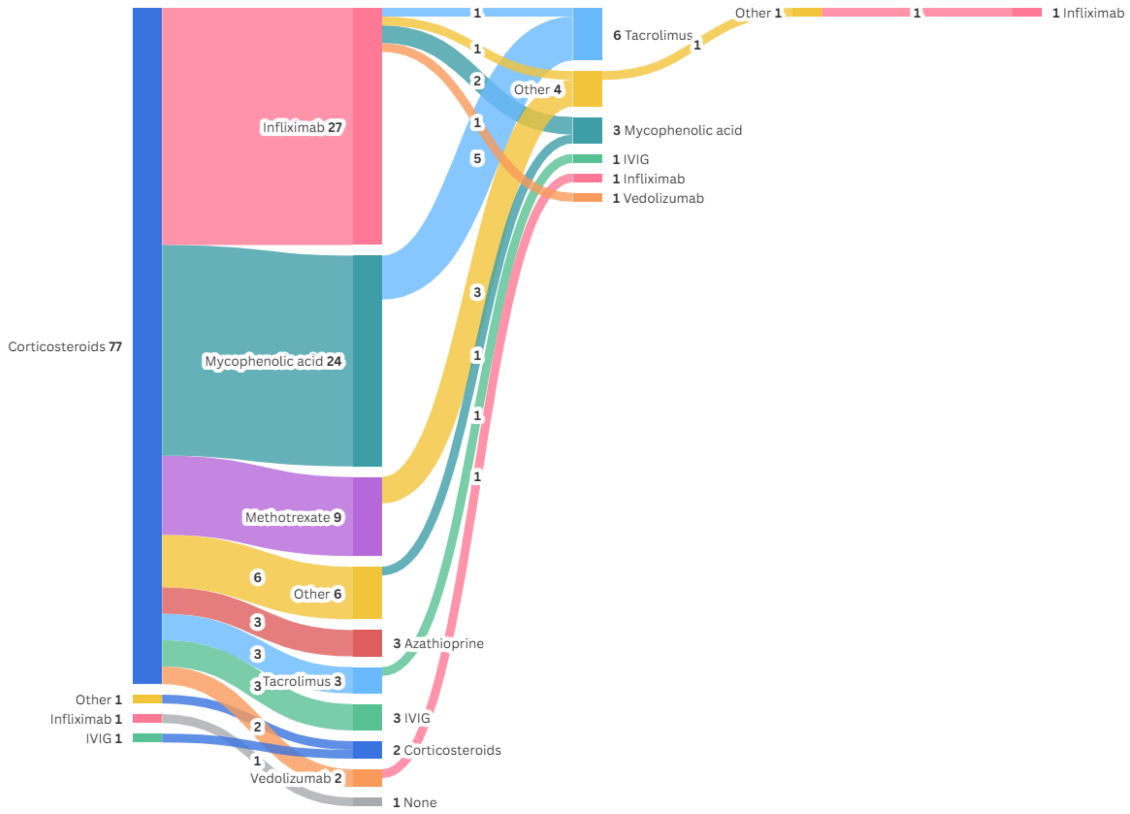

Supplementary Figure S4: Alluvial plot displaying type and administration order of immunosuppressants for patients receiving second-line immunosuppressive treatment (n=80)

Supplementary Table S2: Overview of causes of death in the study population

Cause of death		Number of patients (n=419)
**NSCLC-related**	Progressive NSCLC	187 (44.6%)
**Non-NSCLC-related**	Cardiovascular	10 (2.4%)
	CVA	*5 (1.2%)*
	Myocardial infarction	*2 (0.5%)*
	Other	*3 (0.7%)*
	Infection	11 (2.6%)
	Respiratory	*7 (1.7%)*
	Non respiratory	*4 (1.0%)*
	Euthanasia	3 (0.7%)
	Other malignancy	4 (1.0%)
	Respiratory failure with underlying lung disease (COPD, fibrosis)	4 (1.0%)
	Immune-related adverse event	11 (2.6%)
	Other *	7 (1.7%)
	Unspecified, but not related to NSCLC or toxicity	9 (2.1%)
**Unknown**	Unknown cause of death	29 (6.9%)
**Total number of deaths**		275 (65.6%)

* Other causes of death included complicated pericardial drainage, complicated ERCP procedure, gastrointestinal bleeding, intestinal ischemia, intestinal perforation, multi-morbidity with clinical deterioration, and multi-organ failure with unclear etiology.

Supplementary Table S3: Sensitivity analysis with true corticosteroid peak dose (non-winsorized data).

Multivariable cox proportional hazard regression model assessing the association of corticosteroid peak dose (mg prednisolone equivalent) and second-line immunosuppression with overall survival, cancer-specific survival, and progression-free survival. For corticosteroid peak dose restricted cubic splines were used. Hazard ratios were adjusted for sex, age, ECOG PS, presence of brain metastases, presence of liver metastases, line of anticancer treatment, type of anticancer treatment, and type of irAE.

Variable	HR (95% CI) OS[1]	HR (95% CI) CSS[1]	HR (95% CI) PFS[1]
**Peak dose corticosteroids**			
40mg	—	—	—
80mg	1.28 (1.02-1.62)	1.19 (0.90-1.58)	0.98 (0.74-1.29)
160mg	1.52 (1.08-2.14)	1.36 (0.90-2.04)	1.00 (0.67-1.49)
**Lines of immunosuppression**			
Corticosteroids only	—	—	—
Second-line immunosuppression	0.90 (0.63-1.28)	0.80 (0.51-1.27)	0.72 (0.47-1.11)

[1] HR = Hazard Ratio, CI = Confidence Interval
OS = Overall Survival, CSS = Cancer-specific survival, PFS = Progression-free survival

A

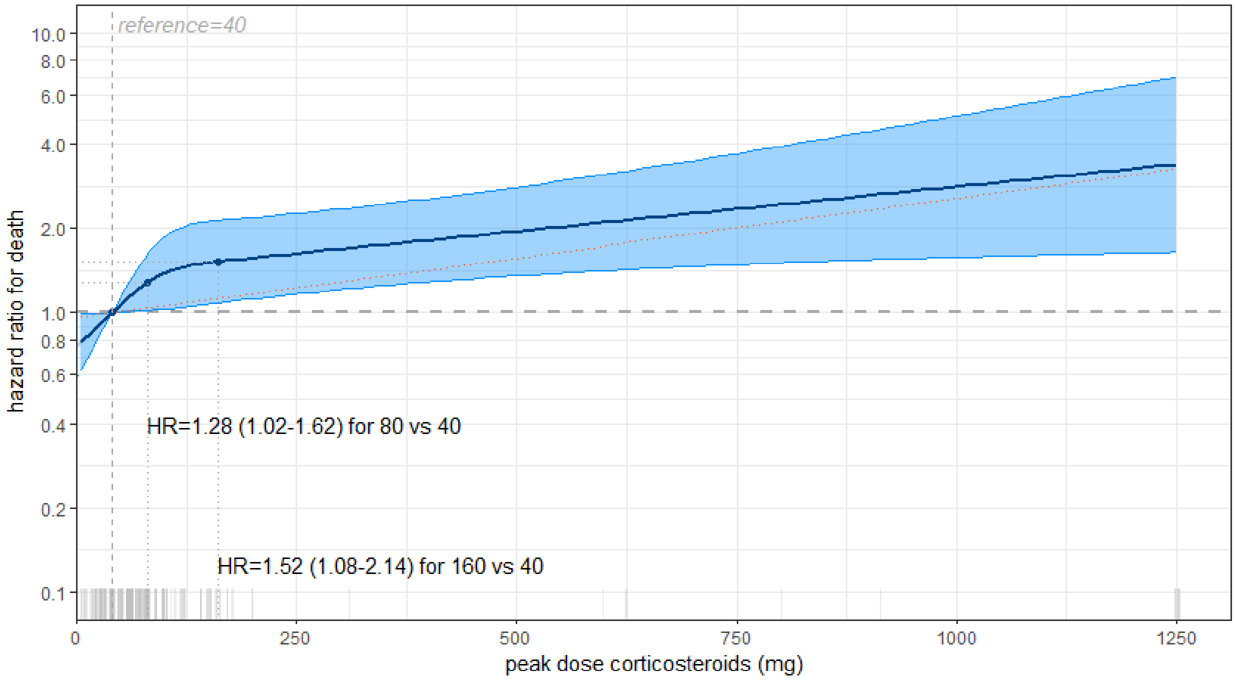

Adjusted for *Lines of immunosuppression, Sex, Age, ECOG PS, Brain metastases, Liver metastases, Line of treatment, Type of treatment*, and *Type of irAE*.

B

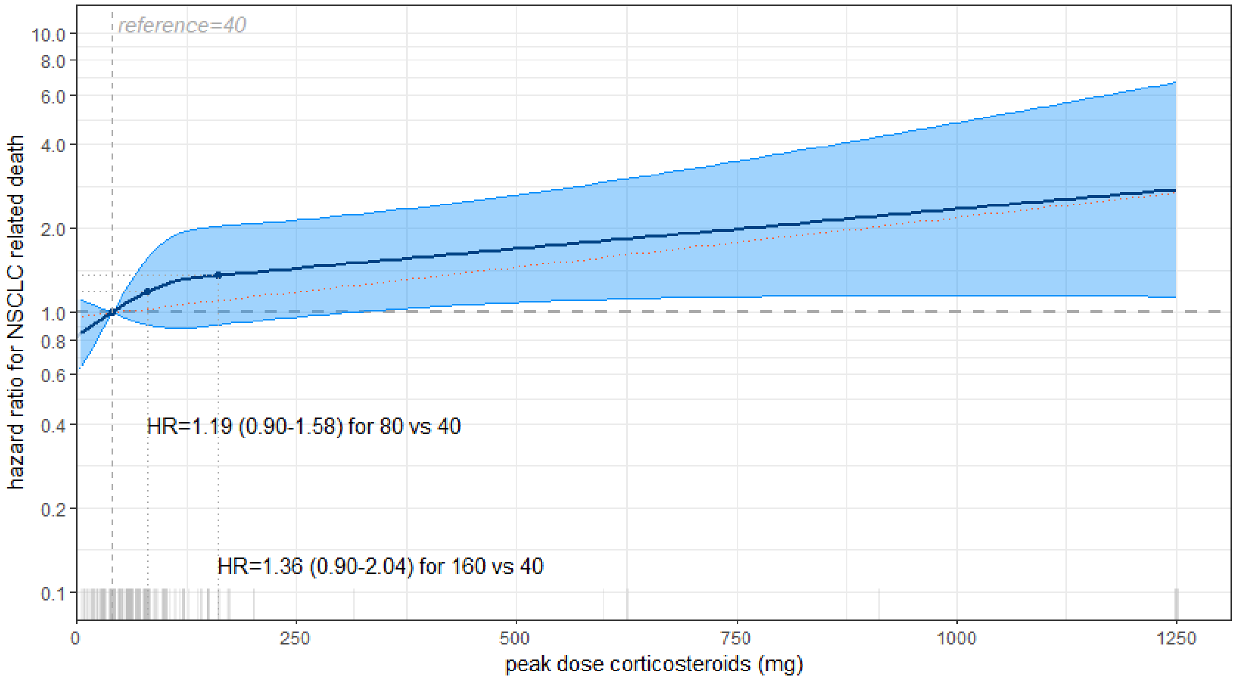

Adjusted for *Lines of immunosuppression, Sex, Age, ECOG PS, Brain metastases, Liver metastases, Line of treatment, Type of treatment*, and *Type of irAE*.

C

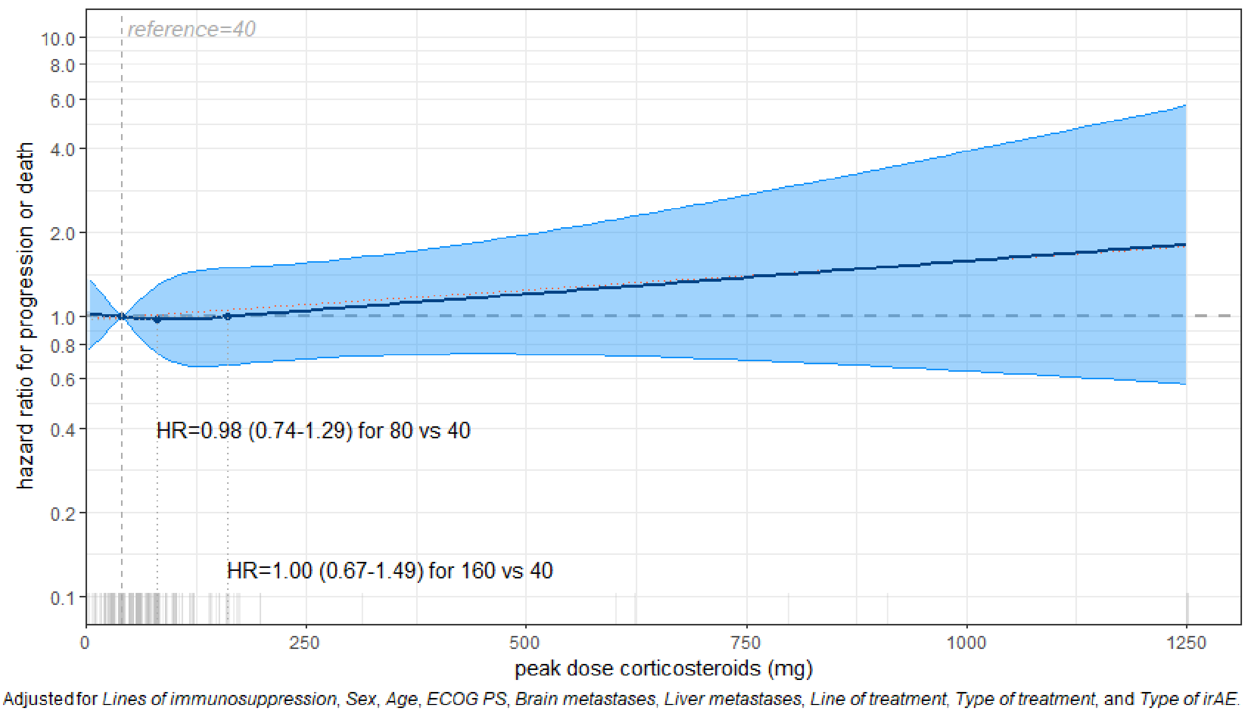

Supplementary Figure S5.

Restricted cubic splines model displaying adjusted hazard ratios for survival for each possible peak dose of corticosteroids relative to 40mg. Hazard ratios were adjusted for second-line immunosuppression, sex, ECOG performance score, line of treatment, age, presence of brain metastases, presence of liver metastases, type of treatment (ICI and concurrent chemotherapy) and type of immune related adverse event. **5A** Hazard ratios for death due to any cause (overall survival); **5B** Hazard ratios for death due to non-small cell lung cancer (cancer-specific survival); **5C** Hazard ratios for progression or death (progression-free survival).

Supplementary Table S4: Association of immunosuppression and survival in subset of patients that was suitable for PFS analyses

Multivariable Cox proportional hazards analysis assessing the association of peak dose corticosteroids and second-line immunosuppression with survival (measured from the start of immunosuppression), excluding patients that had progressive disease prior to the start of immunosuppression.

Variable	HR (95% CI) OS[1]*	HR (95% CI) CSS[1]*
**Peak dose corticosteroids (per 80mg)**	1.44 (1.08-1.93)	1.32 (0.92-1.89)
**Lines of immunosuppression**		
Only corticosteroids	—	—
Second-line immunosuppression	0.99 (0.67-1.46)	0.91 (0.55-1.51)

[1] HR = Hazard Ratio, CI = Confidence Interval
OS = Overall Survival, CSS = Cancer-specific survival, PFS = Progression-free survival

* Hazard ratios for models including corticosteroid peak dose, second-line immunosuppression, sex, age, ECOG PS, presence of brain metastases, presence of liver metastases, line of anticancer treatment, type of anticancer treatment, and type of irAE.

Supplementary Table S5: Association between immunosuppression and survival as measured from the start of ICI therapy

Multivariable Cox proportional hazards analysis assessing the association between peak dose corticosteroids and second-line immunosuppression with survival measured from the start of immune checkpoint inhibition therapy.

Variable	HR (95% CI) OS[1]*	HR (95% CI) CSS[1]*	HR (95% CI) PFS[1]*
**Peak dose corticosteroids (per 80mg)**	1.63 (1.27-2.08)	1.50 (1.12-2.02)	1.11 (0.79-1.55)
**Lines of immunosuppression**			
Corticosteroids only	—	—	—
Second-line immunosuppression	0.86 (0.60-1.23)	0.74 (0.47-1.17)	0.72 (0.47-1.10)

[1] HR = Hazard Ratio, CI = Confidence Interval
OS = Overall Survival, CSS = Cancer-specific survival, PFS = Progression-free survival

* Hazard ratios for models including corticosteroid peak dose, second-line immunosuppression, sex, age, ECOG PS, presence of brain metastases, presence of liver metastases, line of anticancer treatment, type of anticancer treatment, and type of irAE.

Supplementary Table S6: Association between immunosuppression and survival when also including irAE grade in multivariable analyses

Variable	HR (95% CI) OS[1]*	HR (95% CI) CSS[1]*	HR (95% CI) PFS[1]*
**Peak dose corticosteroids (per 80mg)**	1.35 (1.02-1.77)	1.36 (0.97-1.91)	1.04 (0.73-1.47)
**Lines of immunosuppression**			
Corticosteroids only	—	—	—
Second-line immunosuppression	0.88 (0.61-1.25)	0.84 (0.53-1.33)	0.75 (0.48-1.15)
**Grade immune-related adverse event**			
1-2	—	—	—
3	1.52 (1.12-2.06)	1.58 (1.10-2.28)	1.03 (0.72-1.45)
4-5	2.52 (1.60-3.97)	1.24 (0.63-2.43)	1.20 (0.63-2.29)

[1] HR = Hazard Ratio, CI = Confidence Interval
OS = Overall Survival, CSS = Cancer-specific survival, PFS = Progression-free survival

* Hazard ratios for models including corticosteroid peak dose, second-line immunosuppression, sex, age, ECOG PS, presence of brain metastases, presence of liver metastases, line of anticancer treatment, type of anticancer treatment, type of irAE, and grade of irAE.

Supplementary Table S7: Association between immunosuppression and survival in patients with grade 3 irAEs only

Variable	HR (95% CI) OS[1]*	HR (95% CI) CSS[1]*	HR (95% CI) PFS[1]*
**Peak dose corticosteroids (per 80mg)**	1.43 (0.99-2.09)	1.59 (1.04-2.43)	0.92 (0.56-1.52)
**Lines of immunosuppression**			
Corticosteroids only	—	—	—
Second-line immunosuppression	0.95 (0.57-1.60)	1.07 (0.59-1.97)	0.78 (0.42-1.44)

[1] HR = Hazard Ratio, CI = Confidence Interval
OS = Overall Survival, CSS = Cancer-specific survival, PFS = Progression-free survival

* Hazard ratios for models including corticosteroid peak dose, second-line immunosuppression, sex, age, ECOG PS, presence of brain metastases, presence of liver metastases, line of anticancer treatment, type of anticancer treatment, and type of irAE.

Supplementary Table S8: Association between immunosuppression and survival in patients with ICI monotherapy (no concurrent chemotherapy) only

Variable	HR (95% CI) OS[1]*	HR (95% CI) CSS[1]*	HR (95% CI) PFS[1]*
**Peak dose corticosteroids (per 80mg)**	1.64 (1.19-2.26)	1.65 (1.10-2.48)	0.93 (0.59-1.46)
**Lines of immunosuppression**			
Corticosteroids only	—	—	—
Second-line immunosuppression	0.76 (0.48-1.19)	0.60 (0.32-1.10)	0.52 (0.29-0.94)

[1] HR = Hazard Ratio, CI = Confidence Interval
OS = Overall Survival, CSS = Cancer-specific survival, PFS = Progression-free survival

* Hazard ratios for models including corticosteroid peak dose, second-line immunosuppression, sex, age, ECOG PS, presence of brain metastases, presence of liver metastases, line of anticancer treatment, type of anticancer treatment, and type of irAE.

Supplementary Table S9: Multivariable Cox proportional hazards regression analysis with cumulative dose corticosteroids and use of second-line immunosuppressants

Variable	HR (95% CI) OS[1]*	HR (95% CI) CSS[1]*	HR (95% CI) PFS[1]*
**Cumulative dose corticosteroids (per 1000mg)**	0.99 (0.97-1.02)	0.99 (0.97-1.02)	1.00 (0.99-1.02)
**Lines of immunosuppression**			
Corticosteroids only	—	—	—
Second-line immunosuppression	0.91 (0.64-1.30)	0.81 (0.51-1.28)	0.73 (0.48-1.13)

[1] HR = Hazard Ratio, CI = Confidence Interval
OS = Overall Survival, CSS = Cancer-specific survival, PFS = Progression-free survival

* Hazard ratios for models including cumulative corticosteroid dose, second-line immunosuppression, sex, age, ECOG PS, presence of brain metastases, presence of liver metastases, line of anticancer treatment, type of anticancer treatment, and type of irAE.

Supplementary Table S10: Landmark analysis to assess the association between cumulative dose corticosteroids and survival while reducing the effect of immortal time bias

Multivariable Cox proportional hazards analysis assessing cumulative dose corticosteroids and second-line immunosuppression in the subset of patients that was alive after 6 months and that had a maximum duration of corticosteroid therapy of 6 months (n=185 for OS/CSS, n=169 for PFS analyses).

Variable	HR (95% CI) OS[1]*	HR (95% CI) CSS[1]*	HR (95% CI) PFS[1]*
**Cumulative dose corticosteroids (per 1000mg)**	1.04 (0.93-1.16)	1.02 (0.90-1.16)	0.98 (0.88-1.11)
**Lines of immunosuppression**			
Corticosteroids only	—	—	—
Second-line immunosuppression	0.88 (0.41-1.85)	0.88 (0.36-2.14)	0.69 (0.29-1.63)

[1] HR = Hazard Ratio, CI = Confidence Interval
OS = Overall Survival, CSS = Cancer-specific survival, PFS = Progression-free survival

* Hazard ratios for models including cumulative corticosteroid dose, second-line immunosuppression, sex, age, ECOG PS, presence of brain metastases, presence of liver metastases, line of anticancer treatment, type of anticancer treatment, and type of irAE.

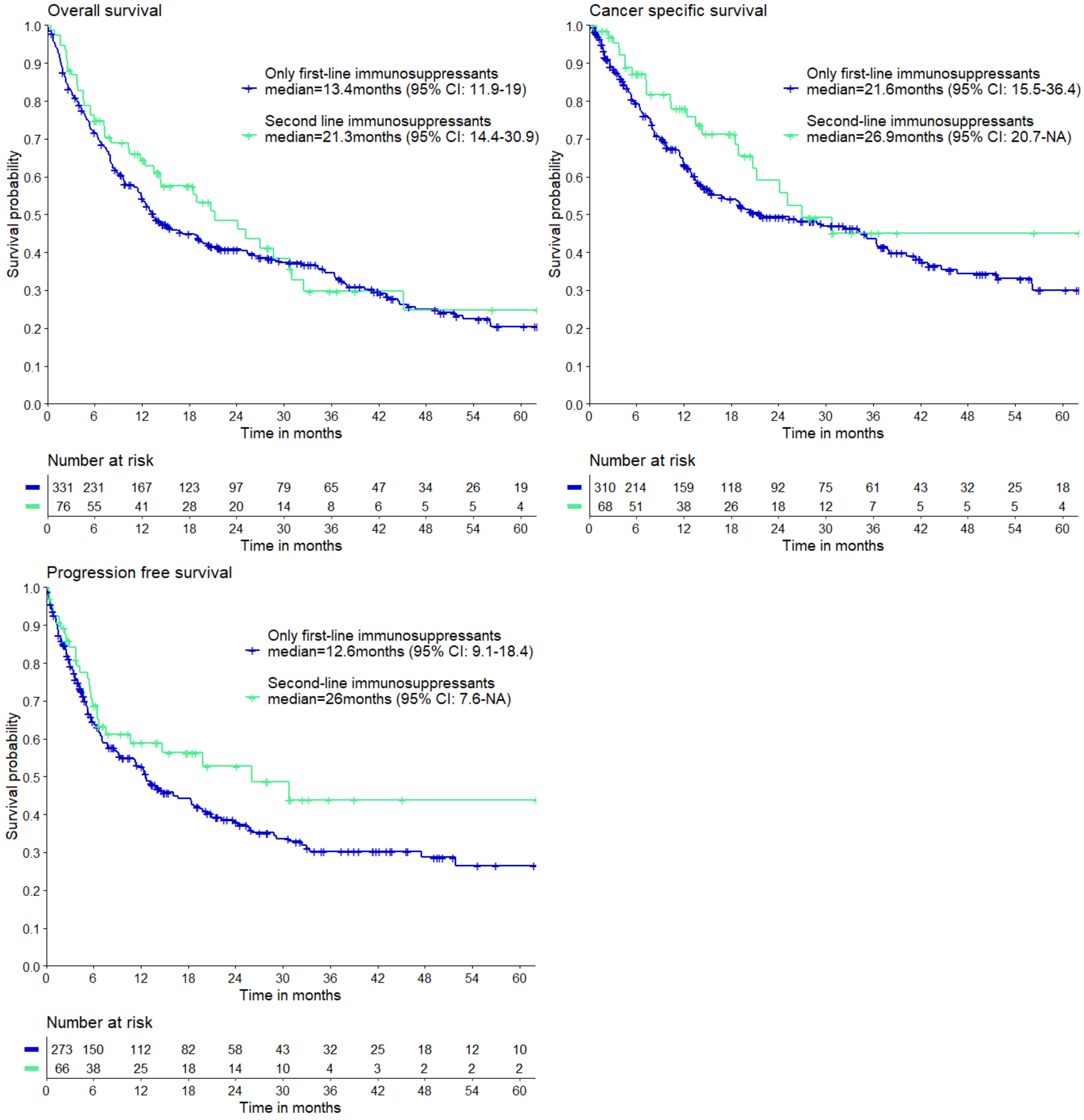

Supplementary Figure S6

Overall survival, cancer specific survival, and progression-free survival as assessed by Kaplan Meier analyses, measured from start immunosuppression, stratified for type of immunosuppression.

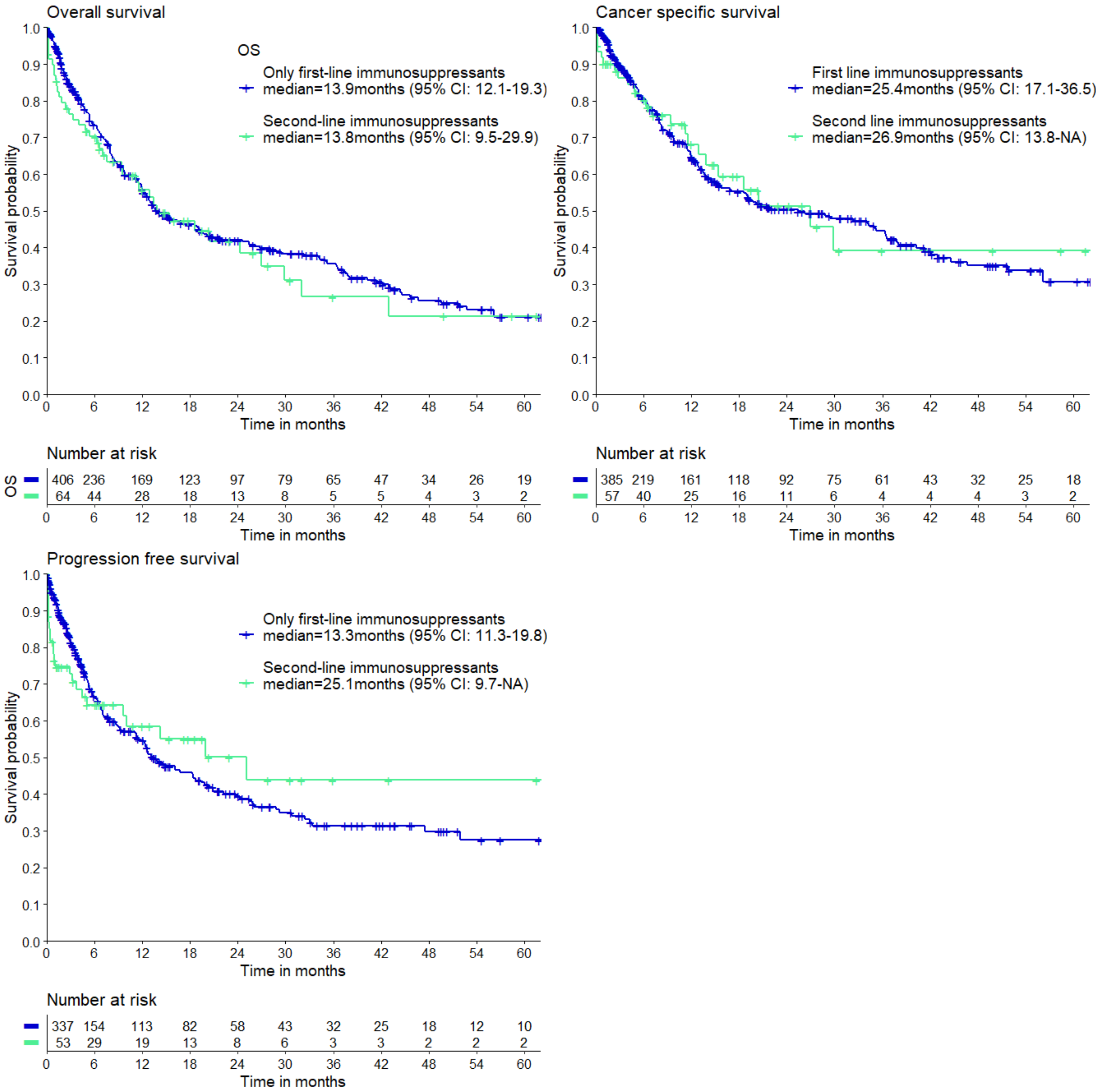

Supplementary Figure S7

Simon-Makuch survival plots† with the start of second-line immunosuppression included as time-varying covariate. For patients with corticosteroids only, survival was measured from start of corticosteroids until progression or death, last follow-up, or start of second-line immunosuppression, whichever occurred first. For patients with second-line immunosuppression, survival was measured from start of second-line immunosuppression until progression, death, or last follow-up.

† *Simon R, Makuch RW. A non-parametric graphical representation of the relationship between survival and the occurrence of an event: Application to responder versus non-responder bias. Statistics in Medicine. 1984;3(1):35-44. Doi: 10.1002/sim.4780030106*

Supplementary Table S11: Multivariable Cox regression analysis in time-varying survival analysis

Variable	HR (95% CI) OS[1]*	HR (95% CI) CSS[1]*	HR (95% CI) PFS[1]*
**Peak dose corticosteroids (per 80mg)**	1.60 (1.25-2.04)	1.52 (1.12-2.06)	1.07 (0.76-1.49)
**Lines of immunosuppression**			
Corticosteroids only	—	—	—
Second-line immunosuppression	1.15 (0.79-1.66)	1.06 (0.66-1.70)	0.99 (0.63-1.54)

[1] HR = Hazard Ratio, CI = Confidence Interval
OS = Overall Survival, CSS = Cancer-specific survival, PFS = Progression-free survival

* Hazard ratios for models including corticosteroid peak dose, second-line immunosuppression, sex, age, ECOG PS, presence of brain metastases, presence of liver metastases, line of anticancer treatment, type of anticancer treatment, and type of irAE.

Supplementary Table S12: Sensitivity analysis adjusting for ICI resumption

Multivariable Cox proportional hazards analysis assessing the association between peak dose corticosteroids and second-line immunosuppression with survival, adjusted for resumption of checkpoint inhibition therapy.

Variable	HR (95% CI) OS[1]*	HR (95% CI) CSS[1]*	HR (95% CI) PFS[1]*
**Peak dose corticosteroids (per 80mg)**	1.53 (1.18-1.98)	1.47 (1.06-2.04)	1.22 (0.85-1.75)
**Lines of immunosuppression**			
Corticosteroids only	—	—	—
Second-line immunosuppression	0.96 (0.65-1.41)	0.93 (0.57-1.52)	0.91 (0.57-1.47)

[1] HR = Hazard Ratio, CI = Confidence Interval
OS = Overall Survival, CSS = Cancer-specific survival, PFS = Progression-free survival

* Hazard ratios for models including corticosteroid peak dose, second-line immunosuppression, sex, age, ECOG PS, presence of brain metastases, presence of liver metastases, line of anticancer treatment, type of anticancer treatment, and type of irAE.

Supplementary Table S13: Sensitivity analysis adjusting for either ICI resumption or ICI rechallenge

Multivariable Cox proportional hazards analysis assessing the association between peak dose corticosteroids and second-line immunosuppression with survival, adjusted for either resumption or rechallenge of checkpoint inhibition therapy.

Variable	HR (95% CI) OS[1]*	HR (95% CI) CSS[1]*	HR (95% CI) PFS[1]*
**Peak dose corticosteroids (per 80mg)**	1.49 (1.17-1.91)	1.44 (1.06-1.95)	1.02 (0.72-1.44)
**Lines of immunosuppression**			
Corticosteroids only	—	—	—
Second-line immunosuppression	0.84 (0.59-1.20)	0.79 (0.50-1.25)	0.76 (0.49-1.18)

[1] HR = Hazard Ratio, CI = Confidence Interval
OS = Overall Survival, CSS = Cancer-specific survival, PFS = Progression-free survival

* Hazard ratios for models including corticosteroid peak dose, second-line immunosuppression, sex, age, ECOG PS, presence of brain metastases, presence of liver metastases, line of anticancer treatment, type of anticancer treatment, and type of irAE.
